# Supplementary material for: Local Exhaust Ventilation to Control Dental Aerosols and Droplets
Source: J Dent Res. 2021 Nov 10;101(4):384–91. doi: 10.1177/00220345211056287 (PMC8935467; doi:10.1177/00220345211056287)
Supplement: sj-docx-1-jdr-10.1177_00220345211056287 – Supplemental material for Local Exhaust Ventilation to Control Dental Aerosols and Droplets [file sj-docx-1-jdr-10.1177_00220345211056287.docx]

**Local Exhaust Ventilation to Control Dental Aerosols and Droplets**

James R Allison, Christopher Dowson, Kimberley Pickering, Greta Červinskytė, Justin Durham, Nicholas S Jakubovics, Richard Holliday

**Supplementary Appendix**

**Methods**

*Pilot testing*

Preliminary testing was carried out to ensure fluorescein was captured by the Local Exhaust Ventilation (LEV) device and not redistributed into the environment which could lead to spurious results. Three filter papers were placed 11 cm away from the two exhaust vents on both sides of the LEV device, and one BioSampler was placed 22 cm away from each vent (total 6 filter papers and 2 BioSamplers). The LEV was switched on, and after four minutes, an air turbine handpiece was operated with fluorescein tracer as described in the main document, with the spray directed into the LEV nozzle. The handpiece was used for six minutes before stopping, and after 10 further minutes the LEV was turned off and samples were collected. A negative control condition with plain water in clean waterlines instead of fluorescein was also conducted and both conditions were conducted in triplicate. Samples were processed as described in the main document.

**Results**

*Pilot testing*

No difference was seen in fluorescein detection between the negative control and when fluorescein tracer was used for pilot experiments, confirming that fluorescein does not pass through the LEV’s HEPA filter. These data are shown in Appendix Table 2 and Appendix Figure 2.

**
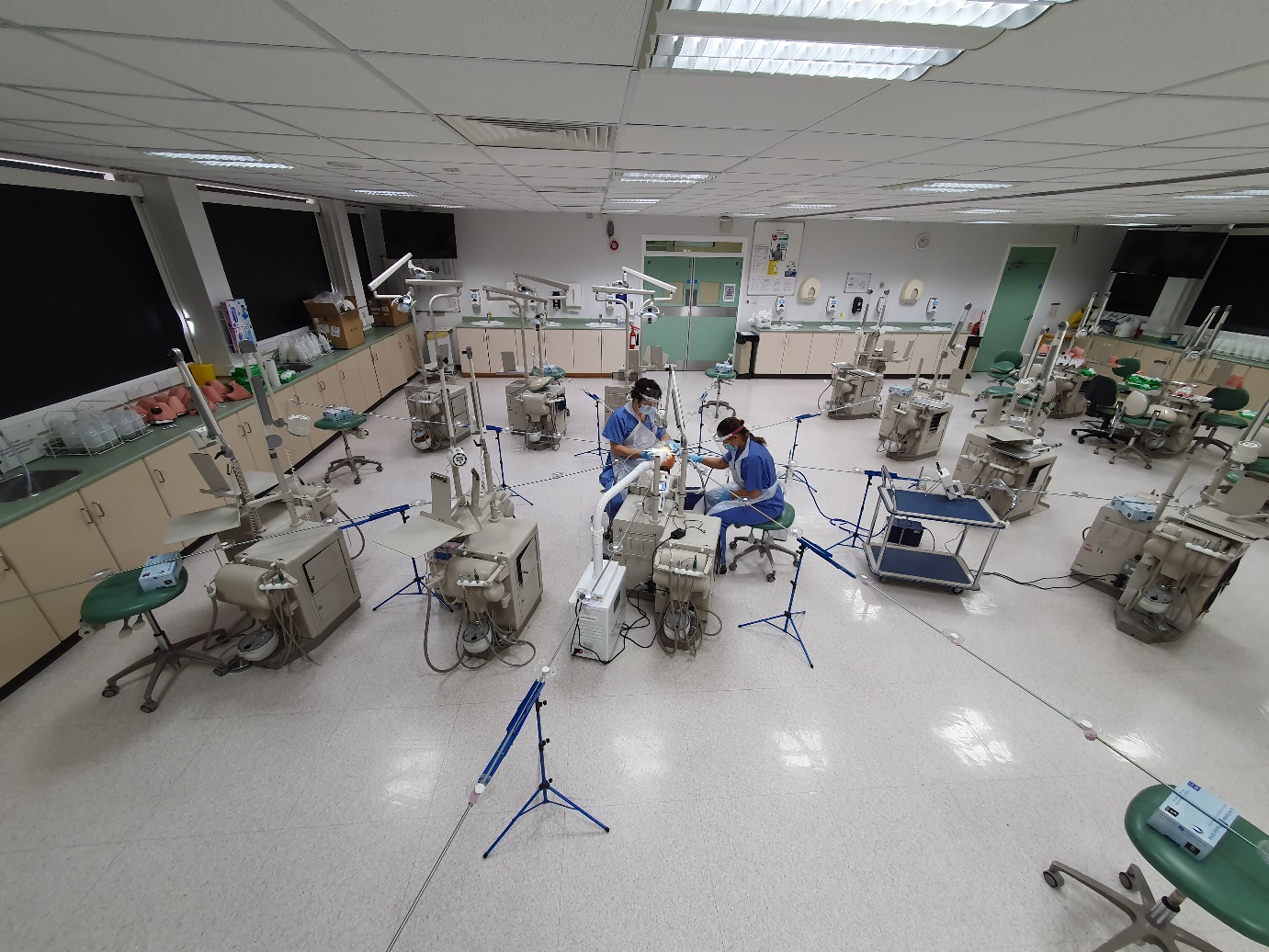
**

**A**

**
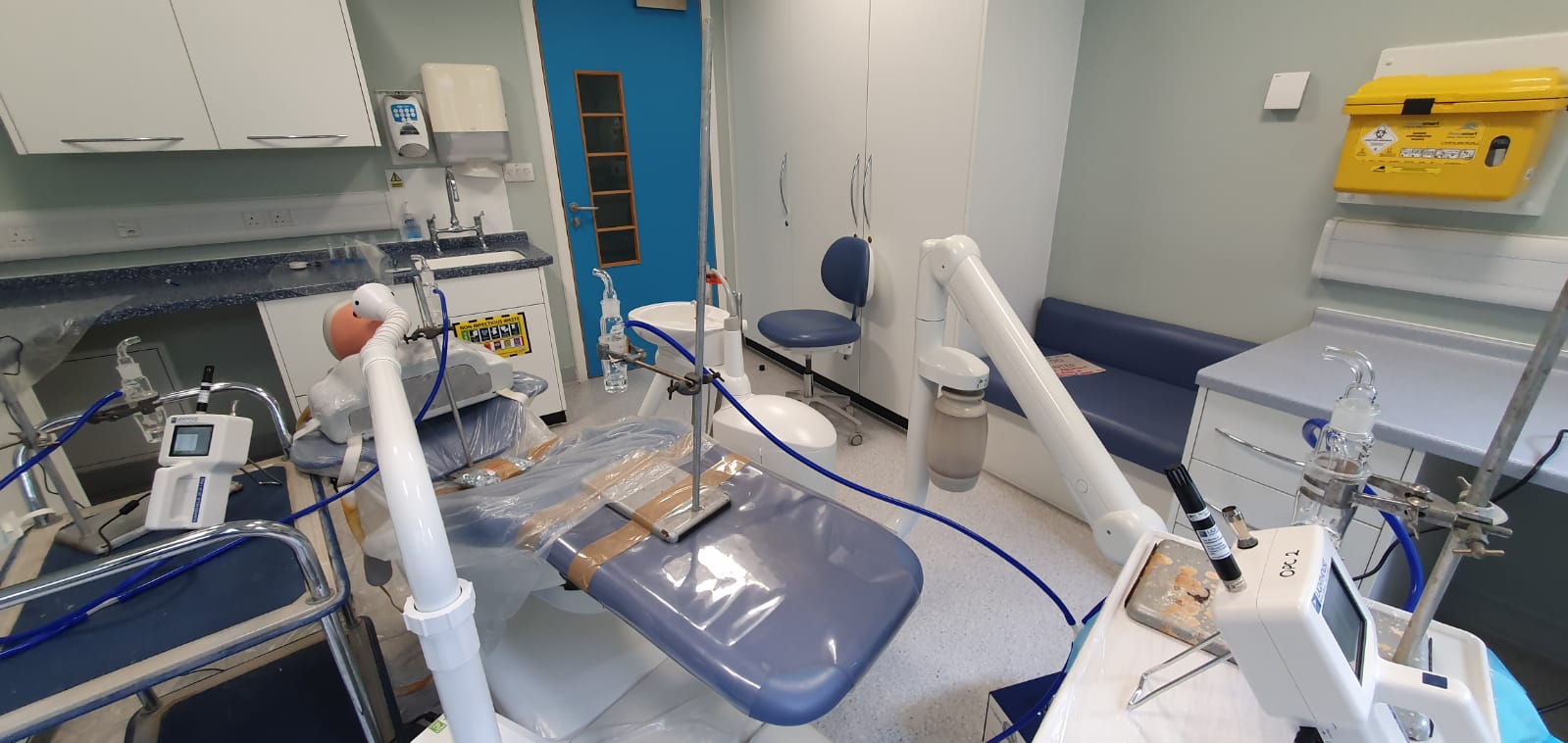
**

**B**

**Appendix Figure 1.** (A) Image of the experimental setup in the open plan setting showing the rig supporting filter papers. BioSamplers and Optical Particle Counter (OPC) number 1 are shown along the arm and trolley in the right of the image, OPC number 2 is adjacent to the LEV nozzle. (B) Image of the experimental setup in the single surgery setting showing BioSamplers placed along the axis of the dental chair, and OPCs placed at the foot of the dental chair and in the left of the image.

| **Open plan setting, 6.5 ACH** | | | | | | | | | |
| --- | --- | --- | --- | --- | --- | --- | --- | --- | --- |
| **Experiment** | **Instrument** | **Procedure** | **Duration, min** | **Suction** | **LEV** | **Irrigant flow rate, mL/ min** | **Measurements (*n* per repetition)** | **Repetitions** |  |
| *LEV* | Air-turbine | Anterior Crown Preparation | 10 | Yes | Yes | 29.3 | BioSampler (4)  OPC (2)  Filter papers (65) | 3 |  |
| *No LEV* | Air-turbine | Anterior Crown Preparation | 10 | Yes | No | 29.3 | BioSampler (4)  OPC (2)  Filter papers (65) | 3 |  |
| *Negative Control* | - | - | 10 | - | - | - | BioSampler (4)  OPC (2)  Filter papers (65) | 3 |  |
|  |  |  |  |  |  |  |  |  |  |
| **Single surgery setting, 5.0 ACH** | | | | | | | | | |
| *Positive control* | Ultrasonic scaler | Full mouth scale | 10 | No | No | 38.6 | BioSampler (4)  OPC (2) | 3 | |
| *Suction only* | Ultrasonic scaler | Full mouth scale | 10 | Yes | No | 38.6 | BioSampler (4)  OPC (2) | 3 | |
| *LEV only* | Ultrasonic scaler | Full mouth scale | 10 | No | Yes | 38.6 | BioSampler (4)  OPC (2) | 3 |  |
| *LEV and suction* | Ultrasonic scaler | Full mouth scale | 10 | Yes | Yes | 38.6 | BioSampler (4)  OPC (2) | 3 |  |
| *Negative Control* | - | - | 10 | - | - | - | BioSampler (4)  OPC (2) | 3 |  |

**Appendix Table 1.** Overview of experiments conducted in each setting. ACH: Air Changes per Hour; LEV: Local Exhaust Ventilation; OPC: Optical Particle Counter.

| **BioSampler (Suspended aerosols)** | | | |  |
| --- | --- | --- | --- | --- |
| **Experiment** | **Mean fluorescence; RFU** | **SD** | ***p*** |  |
| **Negative control**  *n* = 6 | 25.7 | 1.2 | - |  |
| **Fluorescein**  *n* = 6 | 26.2 | 0.7 | 0.240 |  |
|  |  |  |  |  |
| **Filter papers (settled droplets)** | | | | |
| **Experiment** | **Mean fluorescence; RFU** | **SD** | ***p*** |  |
| **Negative control**  *n* = 18 | 113.2 | 91.7 | - |  |
| **Fluorescein**  *n* = 18 | 130.3 | 69.4 | 0.126 |  |

**Appendix Table 2.** Fluorescence readings from pilot testing using filter papers and BioSamplers. The negative control condition used water instead of fluorescein. Data were non-normally distributed, and means were compared using the Man-Whitney U test with exact probabilities. RFU: Relative Fluorescence Units.


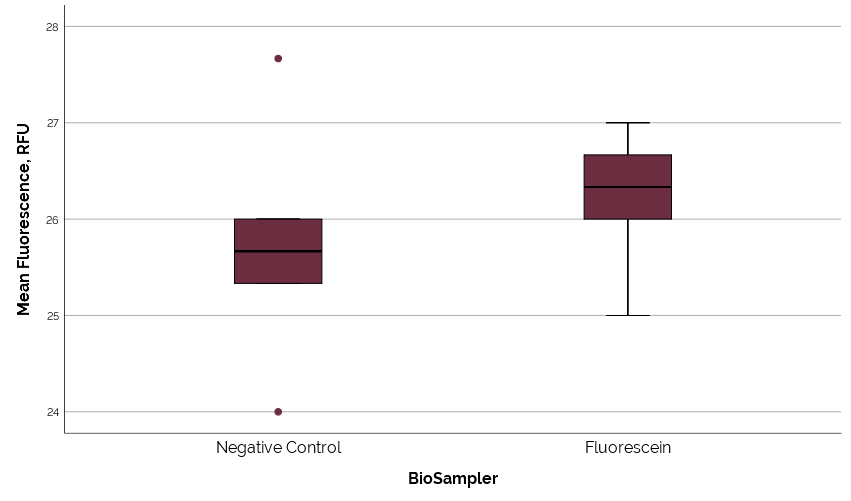

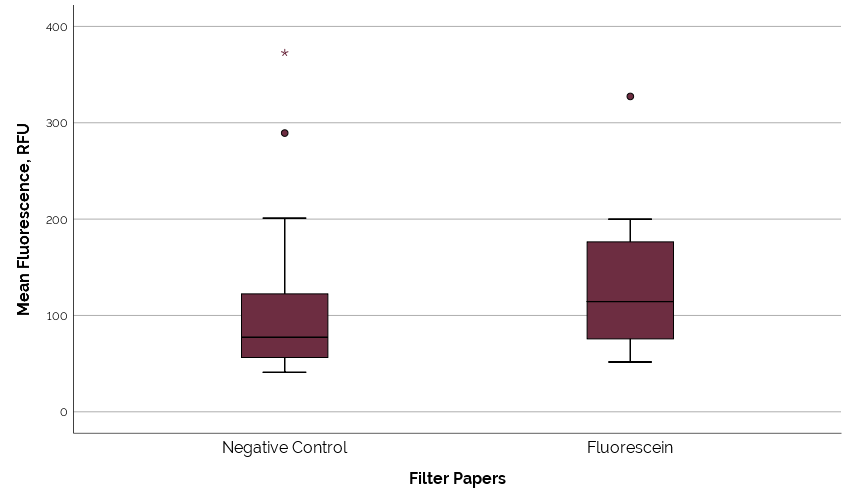


**Appendix Figure 2.** Box plots of fluorescence readings from pilot testing. Relative Fluorescence Units = RFU.

**
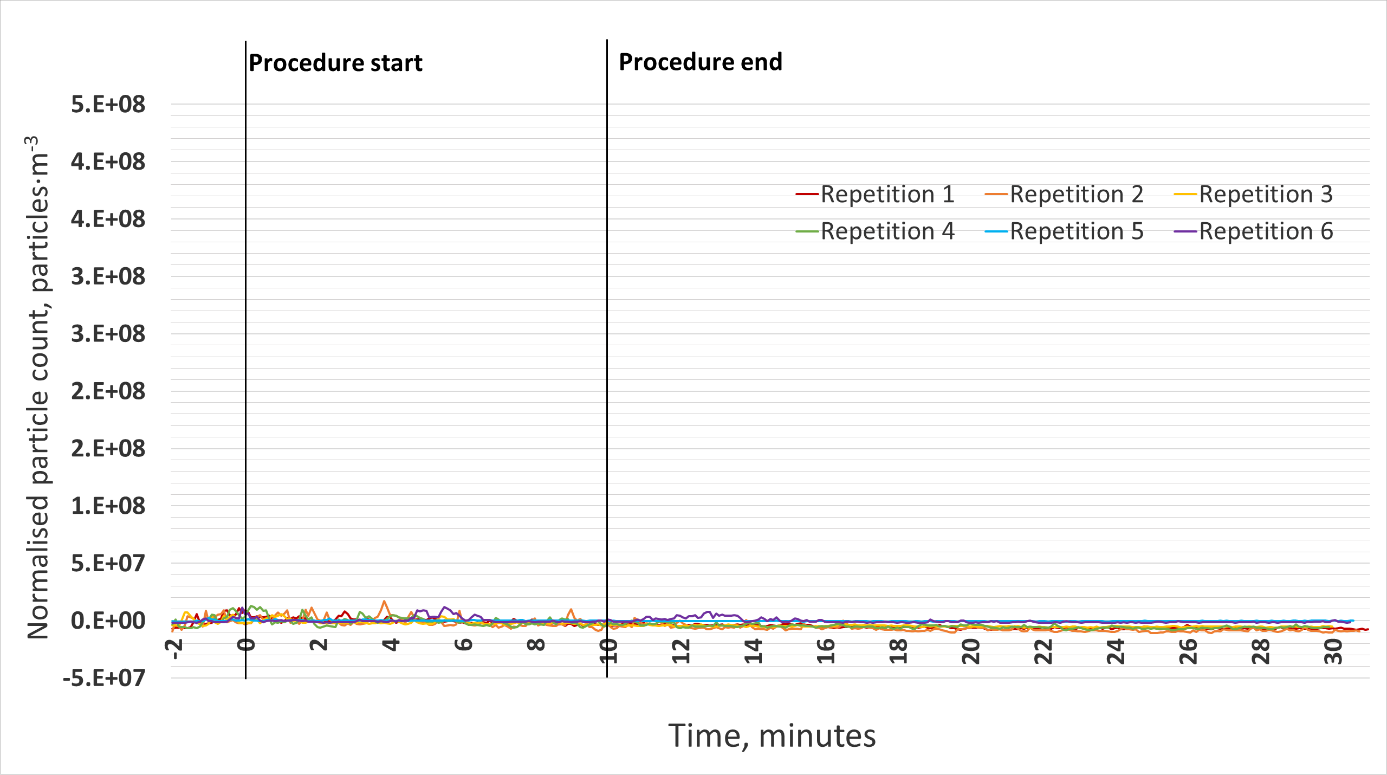
**

**Appendix Figure 3.** Negative control condition (*n* = 6) in the open plan setting where no dental procedure was taking place.

**
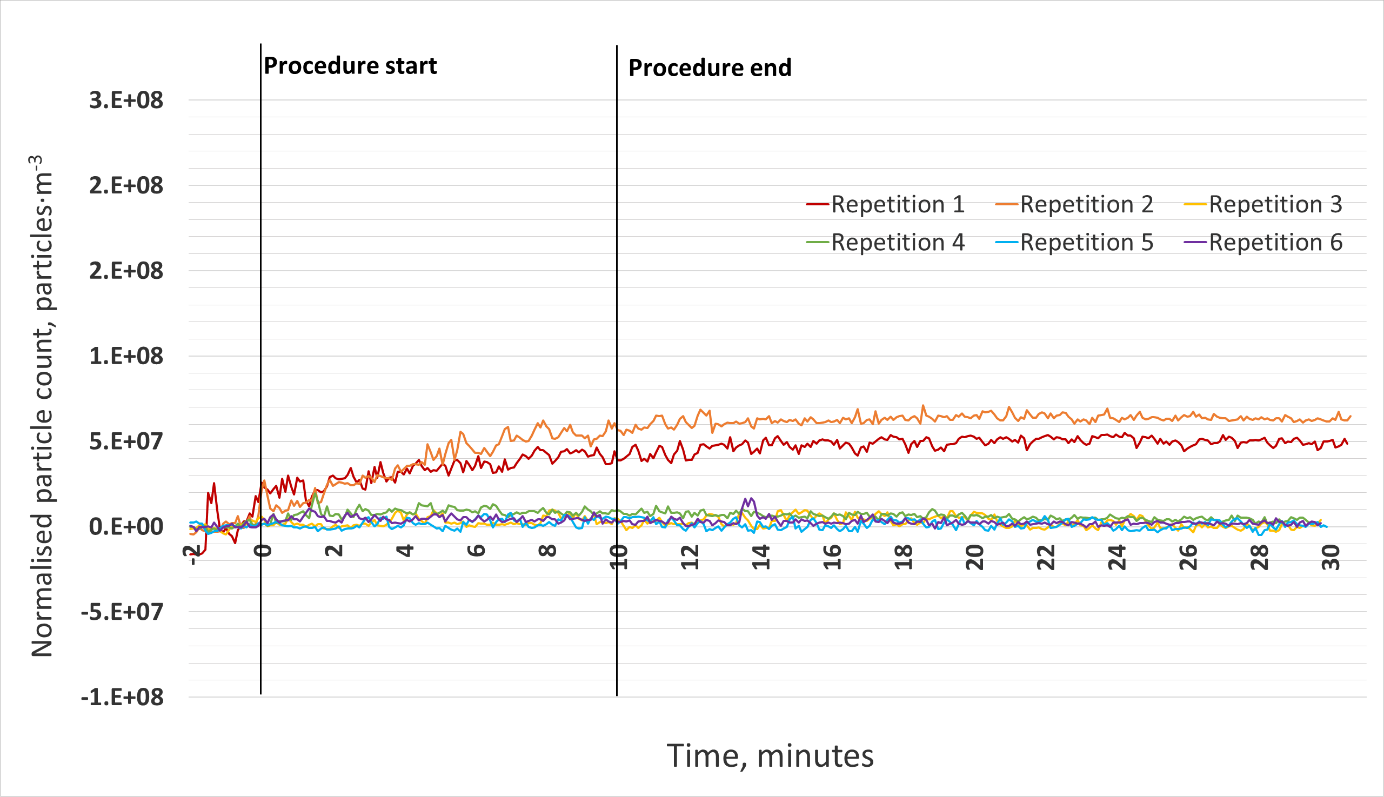
**

**Appendix Figure 4.** Negative control condition (*n* = 6) in the single surgery setting where no dental procedure was taking place. Raised counts in repetitions 1 and 2 likely represent signal drift as instruments equilibrate as these were the first runs.

**
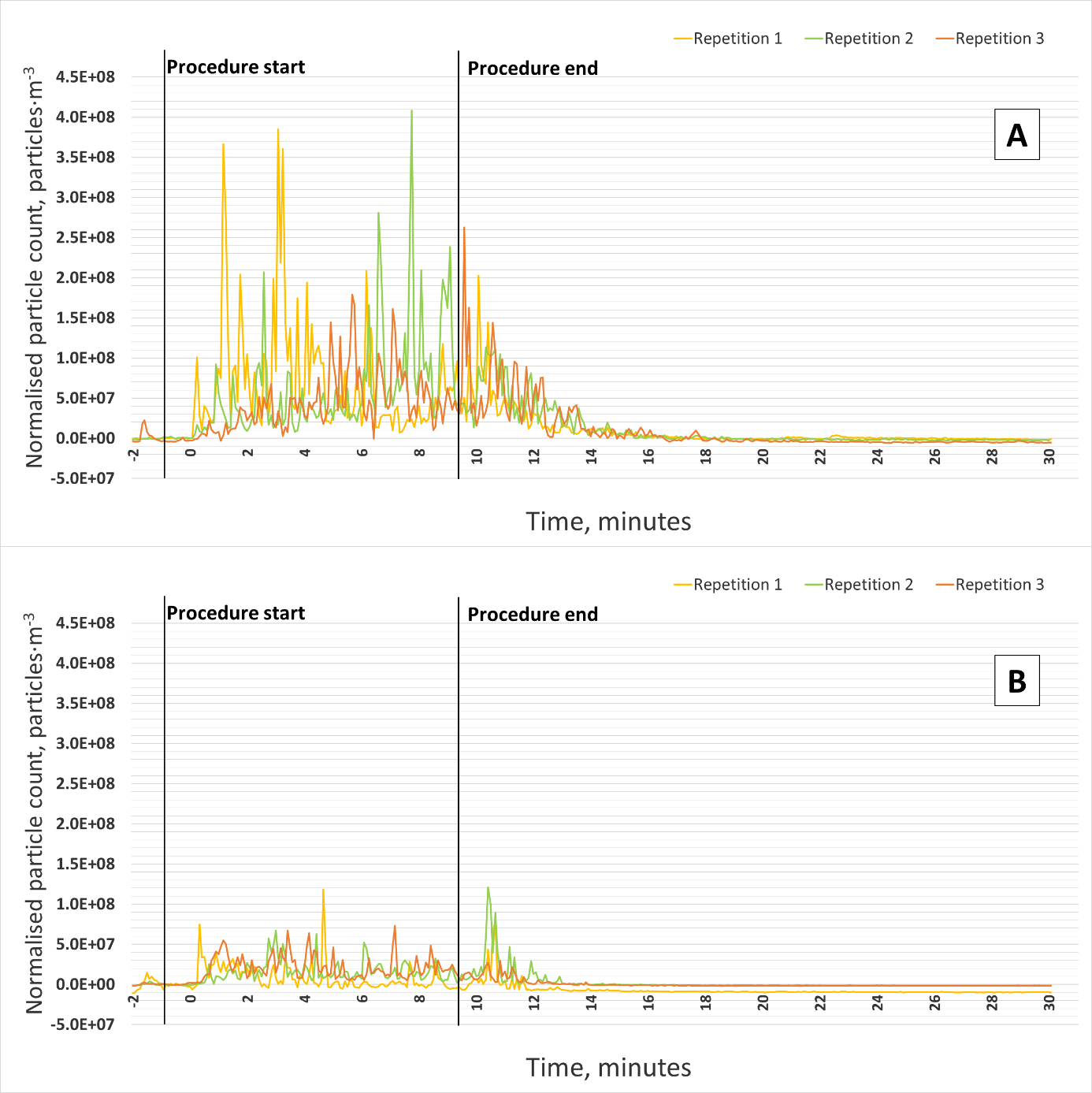
**

**Appendix Figure 5.** Suspended droplets from an air-turbine handpiece as measured by an optical particle counter. Data from three repetitions collected from the 0.5 m sampling position in the open plan setting. A: Positive control (no LEV, with suction); B: LEV


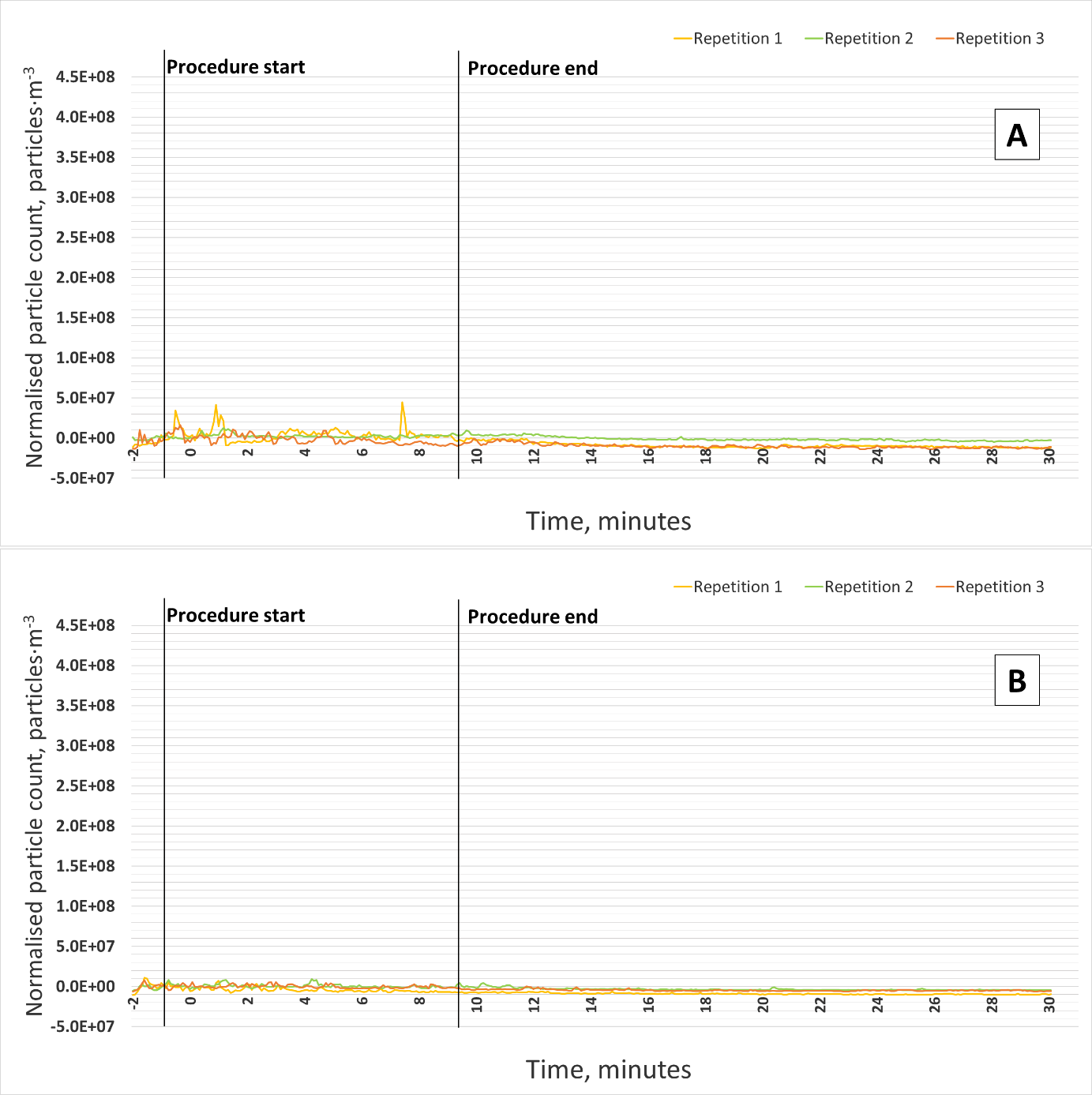


**Appendix Figure 6.** Suspended droplets from an air-turbine handpiece as measured by an optical particle counter. Data from three repetitions collected from the 2 m sampling position in the open plan setting. A: Positive control (no LEV, with suction); B: LEV


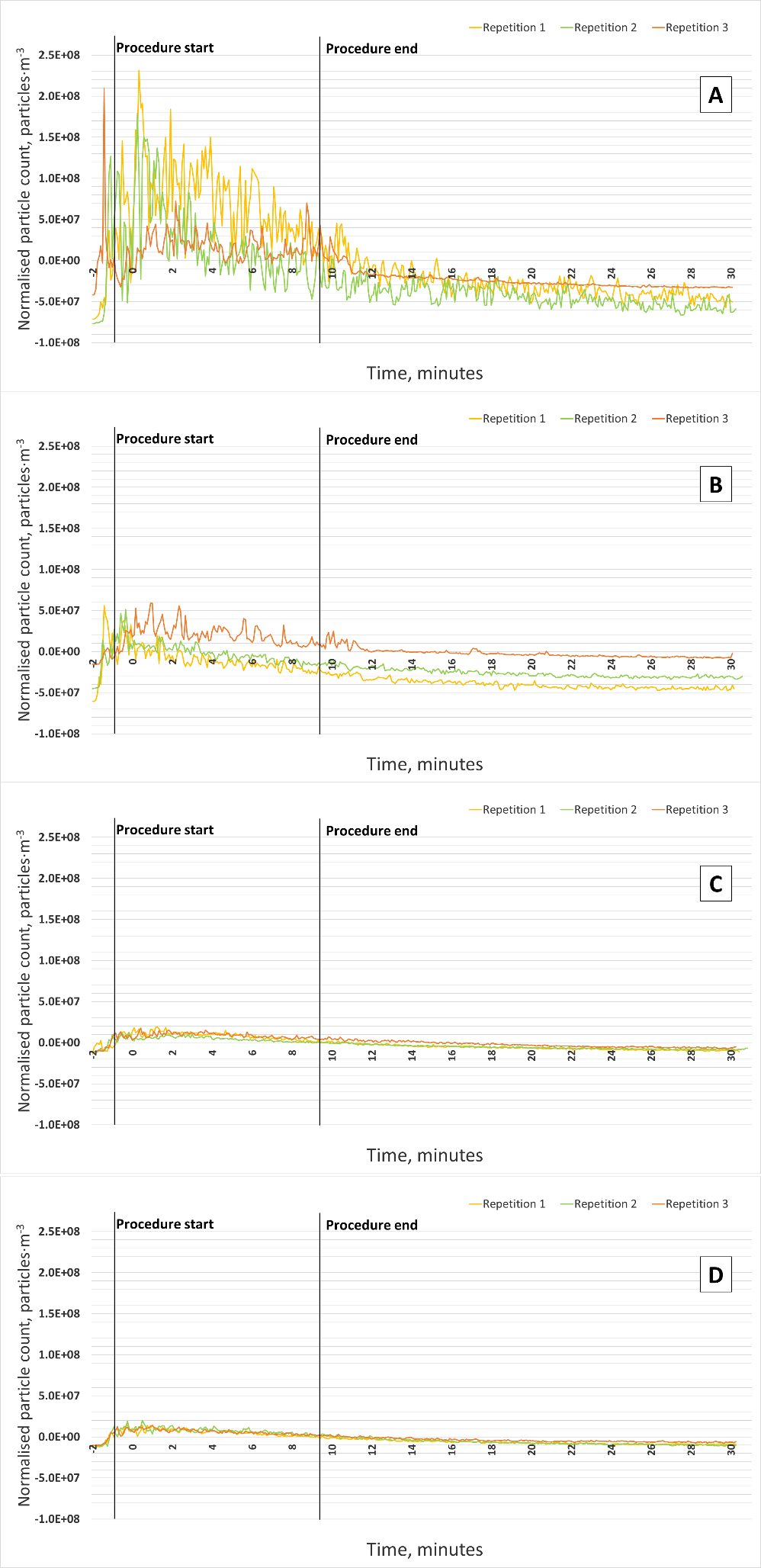


**Appendix Figure 7.** Suspended droplets from an ultrasonic scaler as measured by an optical particle counter. Data from three repetitions collected from the 0.5 m sampling position in the single surgery setting. A: Positive control (no LEV or suction); B: Suction only; C: LEV only; D: LEV and suction.

**
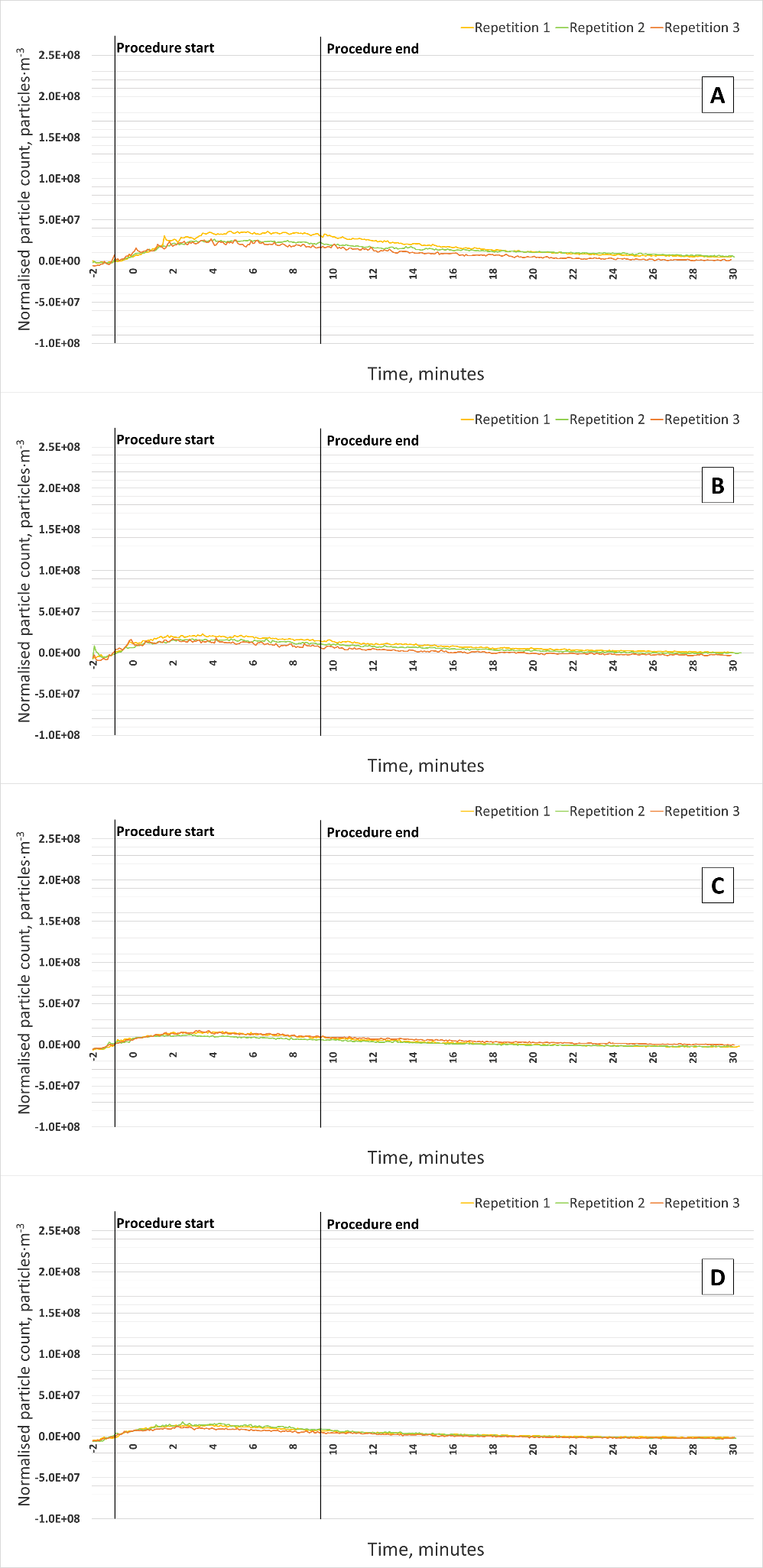
**

**Appendix Figure 8.** Suspended droplets from an ultrasonic scaler as measured by an optical particle counter. Data from three repetitions collected from the 2 m sampling position in the single surgery setting. A: Positive control (no LEV or suction); B: Suction only; C: LEV only; D: LEV and suction.

**Appendix Figure 9.** Fluorescein from the air-turbine handpiece, collected by settlement onto filter paper samples in the open plan setting and measured using fluorometric analysis. Data for each group adjusted for background fluorescence by subtraction of mean negative control values from each sample (≤0.5m = 41 RFU, 1-2m = 41 RFU, 2.5-4m = 39 RFU) before averaging. Error bars are not included as samples were pooled and large variation makes visualisation difficult. Standard deviations are given in Table 2 of the main manuscript. RFU: Relative Fluorescence Units.
